# A Novel Deoxynivalenol-Activated Wheat *Arl6ip4* Gene Encodes an Antifungal Peptide with Deoxynivalenol Affinity and Protects Plants against *Fusarium* Pathogens and Mycotoxins

**DOI:** 10.3390/jof7110941

**Published:** 2021-11-06

**Authors:** Gang Liu, Dong-Yun Zuo, Peng Yang, Wei-Jie He, Zheng Yang, Jing-Bo Zhang, Ai-Bo Wu, Shu-Yuan Yi, He-Ping Li, Tao Huang, Yu-Cai Liao

**Affiliations:** 1 Molecular Biotechnology Laboratory of Triticeae Crops, Huazhong Agricultural University, Wuhan 430070, China; liugangforever@webmail.hzau.edu.cn (G.L.); zdy041@163.com (D.-Y.Z.); yangpeng87@webmail.hzau.edu.cn (P.Y.); heweijie@webmail.hzau.edu.cn (W.-J.H.); xiaobei02@163.com (Z.Y.); jingbozhang@mail.hzau.edu.cn (J.-B.Z.); shuyuanyi@mail.hzau.edu.cn (S.-Y.Y.); hepingli@mail.hzau.edu.cn (H.-P.L.); 2 College of Plant Science and Technology, Huazhong Agricultural University, Wuhan 430070, China; 3 State Key Laboratory of Cotton Biology, Institute of Cotton Research of the Chinese Academy of Agricultural Sciences, Anyang 455000, China; 4 College of Life Science and Technology, Huazhong Agricultural University, Wuhan 430070, China; 5 Key Laboratory of Food Safety Research Institute for Nutritional Sciences, Shanghai Institutes for Biological Sciences, Chinese Academy of Sciences, Shanghai 200031, China; abwu@sibs.ac.cn; 6 Forestry and Fruit Tree Research Institute, Wuhan Academy of Agricultural Sciences, Wuhan 430070, China

**Keywords:** deoxynivalenol, *Fusarium* spp., *Triticum aestivum*, antifungal peptide, resistance, plant–pathogen interactions

## Abstract

Deoxynivalenol (DON) is one of the most widespread trichothecene mycotoxins in contaminated cereal products. DON plays a vital role in the pathogenesis of *Fusarium graminearum*, but the molecular mechanisms of DON underlying *Fusarium*–wheat interactions are not yet well understood. In this study, a novel wheat ADP-ribosylation factor-like protein 6-interacting protein 4 gene, *TaArl6ip4,* was identified from DON-treated wheat suspension cells by suppression subtractive hybridization (SSH). The qRT-PCR result suggested that *TaArl6ip4* expression is specifically activated by DON in both the *Fusarium* intermediate susceptible wheat cultivar Zhengmai9023 and the *Fusarium* resistant cultivar Sumai3. The transient expression results of the TaARL6IP4::GFP fusion protein indicate that *TaArl6ip4* encodes a plasma membrane and nucleus-localized protein. Multiple sequence alignment using microscale thermophoresis showed that TaARL6IP4 comprises a conserved DON binding motif, _67_HXXXG_71_, and exhibits DON affinity with a dissociation constant (*K*_D_) of 91 ± 2.6 µM. Moreover, TaARL6IP4 exhibited antifungal activity with IC_50_ values of 22 ± 1.5 µM and 25 ± 2.6 µM against *Fusarium graminearum* and *Alternaria alternata*, respectively. Furthermore, TaArl6ip4 interacted with the plasma membrane of *Fusarium graminearum* spores, resulting in membrane disruption and the leakage of cytoplasmic materials. The heterologous over-expression of *TaArl6ip4* conferred greater DON tolerance and *Fusarium* resistance in *Arabidopsis.* Finally, we describe a novel DON-induced wheat gene, *TaArl6ip4*, exhibiting antifungal function and DON affinity that may play a key role in *Fusarium–*wheat interactions.

## 1. Introduction

*Fusarium* head blight (FHB) is a globally devastating fungal disease in wheat and barley mainly caused by *Fusarium graminearum* (*F. graminearum*) [1,2,3]. FHB epidemics can not only cause yield losses of grain but also indirectly cause a decline in quality because the fungus contaminates grain with various trichothecene mycotoxins, especially deoxynivalenol (DON) [4,5]. Since the 1990s, FHB has reappeared as a severe agricultural issue in North America, Europe, and China [4,6,7,8]. Breeding progress is hampered by limited sources for FHB resistance, which is a quantitative trait controlled by many minor-effect QTLs in wheat [9,10]. Therefore, it is of importance to explore and exploit potential genetic sources for breeding new FHB-resistant cultivars.

DON is one of the most predominant mycotoxins encountered in grain fields [11]. The epoxide group C12/C13 and the hydroxyl group on C3 are responsible for the toxicity of DON by binding the 60S subunit and inhibiting the formation of peptide bonds during protein synthesis in eukaryotes [12,13,14]. Low exposure of DON causes a reduction in food consumption, while an overdose results in vomiting in animals [15,16]. DON is also phytotoxic at very low concentrations, causing wilting, chlorosis, necrosis, and other symptoms in a wide variety of plants [17]. Interestingly, DON is also well known as a virulence factor, facilitating disease spread within wheat spikes [18,19,20]. However, the biological role of DON in *Fusarium*–wheat interactions is not well elucidated. The catalytic residues of DON-affinity proteins are highly conserved [21]. TRI101 has been reported as a trichothecene 3-O-acetyltransferase in *F. graminearum* and *Fusarium sporotrichioides* (*F. sporotrichioides*), which can catalyze and introduce an acetyl functional group from acetyl coenzyme A into the C3 hydroxyl moiety of DON. Furthermore, a catalytically important _156_HXXXD_160_ motif and a structurally important DFGWG motif in FgTRI101 relative to CoA and DON have been emphasized [22]. Three DON-affinity binders with the HXXXD motif have been characterized from phage display library-based heavy chain antibodies (VHHs), and amino acid sequences derived from three clones have been submitted to GenBank with accession numbers AEI91798.1, AEI91799.1, and AEI91780.1 [21,23]. A similar motif, HXXXD/G, has been found to be functionally important for these DON-interacting proteins.

Successful higher plants have evolved a complex defensive system to avoid biotic stress such as pathogen infections from fungi, bacteria, and viruses, and abiotic stressors such as cold, drought, salinity, and low or high temperatures [24,25]. Plant antimicrobial peptides (AMPs) are known as a group of proteins with molecular weights ranging from 5 to 10 kDa, comprising an essential component of the plant immune system and protecting host plants against pathogens and pests. Major groups of AMPs include thionin, defensin, hevein, lipid transfer proteins (LTPs), and snakin [25,26,27]. The first two thionin-like polypeptides γ1- and γ2-purothionin have been characterized and purified from wheat endosperm [28]. Wheat 4-Cys antimicrobial peptides Tk-AMP-X1 and Tk-AMP-X2 show a spore germination inhibitory effect against *F. graminearum* and *Fusarium verticillioides* at an IC_50_ of 7.5–15 µg mL^−1^ [29]. Defensin MtDef5 has been identified in a model legume, *Medicago truncatula*, inhibiting *F. graminearum* and *Neurospora crassa* fungal growth at submicromolar levels [30]. The overexpression of wheat lipid transfer protein TdLTP4 in *Arabidopsis* results in increased abiotic stress tolerance to NaCl, ABA, JA, and H_2_O_2_ [31]. 

ADP-ribosylation factors (ARFs) are a group of small GTP-binding proteins belonging to the Ras superfamily that regulates a wide range of biological processes and signaling pathways in eukaryotes [32]. These low-molecular-weight proteins can be further categorized into ARFs and ARF-like (ARL) proteins according to their sequence homology and biological functions [33]. To date, ARFs have been widely identified in plant species, including *Arabidopsis* [34,35], potato [36], rice [37,38], wheat [39], and tobacco [40]. ADP-ribosylation factor 6 (ARF6), one of the best characterized ARFs, is a small guanine nucleotide-binding protein that stimulates cholera toxin activation, plays a vital role in vesicular trafficking, and acts as an activator of phospholipase D in eukaryotic cells [41,42]. ARF6 localizes to the plasma membrane in its GTP state and is involved in vesicular trafficking and plasma membrane recycling pathways that lead to cytoskeletal rearrangements [43,44,45,46]. ADP-ribosylation factor-like protein 6-interacting protein 4 (ARL6IP4), also known as SRrp37, is a nucleolus and nucleus speckle-localized protein, which interacts with ARL6 and modulates alternative pre-mRNA splicing with either 5’ distal site activation or the preferential use of the 3’ proximal site in *Homo sapiens* [47]. Homologous proteins of ARL6IP4 from mice, rats, and zebrafish have been identified and predicted to be involved in mRNA processing by their sequence similarities with SRrp37. In addition, SRrp37 may act as a splicing inhibitor of herpes simplex virus I (HSVI) pre-mRNA [48,49]. Although different plant ARL6IP4 proteins have been annotated by the automated computational Gnomon method, no biological function analyses have been conducted. Similarly, no biological function analyses have been conducted for ARL6IP4 genes in wheat thus far.

The present study was conducted to identify new FHB resistance source genes associated with DON stimulation. A novel gene *TaArl6ip4* was identified from a DON-treated wheat suspension cell culture by suppression subtractive hybridization (SSH). We aimed to clone and characterize wheat *TaArl6ip4* to understand its subcellular localization and biological functions in plants. In general, the wheat *TaArl6ip4* gene is a promising genetic resource for FHB resistance breeding and can be utilized in subsequent basic research for revealing the mechanism behind *Fusarium*–wheat interactions.

## 2. Materials and Methods

### 2.1. Plant and Microbial Materials

The *Fusarium* intermediate susceptible wheat cultivar Zhengmai9023 (Z9), *Fusarium* resistant wheat cultivar Sumai3 (S3), and *Arabidopsis thaliana* ‘Columbia’ were used in this study. Z9 is a weak spring bread wheat cultivar that has been widely grown in the middle of the Yangtze River valley; S3 is a spring wheat cultivar that has not been commercially grown due to its poor agronomy characters. *Arabidopsis* and wheat plants were cultivated in a growth chamber under a 16/8-h light/dark cycle at 22 °C, as previously described [50]. Fungal pathogen *F. graminearum* strain 5035 was previously isolated from a scabby wheat spike in Wuhan, China [51], and mutant strain *Tri5¯* with a *Tri5* gene knockout in the background of *F. graminearum* 5035 [52] was cultured on potato dextrose agar (PDA) medium at 28 °C. Suspension cell cultures derived from Z9 wheat seedlings were cultured in an orbital incubator at 150 rpm in the dark at 21 °C, as previously described [53]. *Agrobacterium tumefaciens* strain GV3101, the pTRA vector, and the green fluorescence protein (GFP) gene were used as previously described [54]. A pGEX-6P-1 (GenScript, Piscataway, NJ, USA) GST expression vector was used for recombinant protein prokaryotic expression. Gene transient expression was performed using a PDS-1000 He biolistic gun (Bio-rad laboratories, Hercules, CA, USA) at a pressure of 1100 psi.

### 2.2. RNA Extraction and Suppression Subtractive Hybridization

Seven-day-old Z9 fresh suspension cell cultures were treated with 50 µg mL^−1^ of DON, and sterilized double-distilled water was used as the control. Then, 2 mL of automatically precipitated cells was collected at 4-, 12-, and 48 h post-inoculation (hpi). The mRNAs of the suspension cell cultures after treatment with DON or water at different time intervals were extracted, and suppression subtractive hybridization was performed as previously described [50]. In brief, the mRNAs were isolated from the DON-treated Z9 suspension cell cultures (tester) or the water-treated suspension cell (driver) with a PolyA mRNA Isolation Kit (Promega, Madison, WI, USA), and then 2 µg aliquots of each mRNA were used to generate the cDNA using a PCR-Select cDNA Subtraction Kit (Clontech, San Francisco, CA, USA). Then, after the cDNAs were digested with *Rsa*I, adaptors were added to the end of the derived fragments, and SSH was performed as previously described [55]. The PCR fragments from the SSH were finally constructed into pGEM-T Easy vectors (Promega, Madison, WI, USA).

### 2.3. DON Treatment and Fungal Inoculation of Wheat Spikes

DON treatment and *F. graminearum* inoculation of the wheat spike experiments were carried out as previously described [50]. DON (Sigma–Aldrich, St. Louis, MO, USA) was adjusted to a final concentration of 1.5 mg mL^−1^ with a 0.2% Tween-20 solution; the macroconidial suspension of *F. graminearum* 5035 or the *Tri5¯* strains was adjusted to a working concentration of 5 × 10^5^ spores mL^−1^ in a 0.2% Tween-20 solution. In total, 20 spikes of Z9 and S3 were inoculated with 15 µL of the DON solution or 10 µL of the macroconidial suspension of DON-producing strain *F. graminearum* 5035 or the DON non-producing strain *Tri5¯* by single-floret injection at the anthesis, respectively [52]. The same volume of 0.2% Tween-20 solution was used as the control for each treatment. Then, the treated wheat plants were cultivated under high humidity in a greenhouse for three days under a 16/8-h light/dark cycle at 22 °C. The spikes treated with DON were sampled at 4, 12, 24, and 48 hpi, while the pathogen-inoculated spikes were sampled at 24, 48, 72, and 96 hpi. The relative expression levels of the *TaArl6ip4* gene were then analyzed by qRT-PCR with primer sets *TaArl6ip4*-F5/*TaArl6ip4*-R5 and *Actin*-F/*Actin*-R (Table S1).

### 2.4. Molecular Cloning and Sequence Analysis

A 466-bp cDNA fragment was isolated from the DON-induced up-regulated SSH cDNA library and sequenced. Primer sets were designed according to the 466-bp sequence to amplify the wheat *TaArl6ip4* gene using a cDNA template from DON-treated wheat Z9 spikes at 24 hpi. The rapid amplification of cDNA ends (RACE) approach was applied to obtain the missing 5′- and 3′- sequences of the *TaArl6ip4* gene using a SMART RACE cDNA Amplification Kit (Takara, Dalian, China) using the primer sets listed in Table S1. Then, the full-length cDNAs of the *TaArl6ip4* gene from wheat Z9 and S3 were amplified with the *TaArl6ip4*-F3 and *TaArl6ip4*-R3 primer sets (Table S1). The PCR products were finally constructed into the pGEM-T Easy clone vector and then sequenced (Sangon, Shanghai, China). The CDS region of *TaArl6ip4* was introduced into a pGEX-6P-1 (GenScript, Piscataway, NJ, USA) GST expression vector for prokaryotic expression by the homologous recombination approach using primer sets *TaArl6ip4*-F6 and *TaArl6ip4*-R6 (Table S1).

Assembled gene function was annotated by blastp (protein–protein BLAST) (https://blast.ncbi.nlm.nih.gov/Blast.cgi/ (accessed on 14 September 2021)) with the deduced TaARL6IP4 amino acid sequence. Multiple sequence alignment of the deduced TaARL6IP4 amino acid sequence from Z9 was carried out, together with synthesized DON-affinity binders and TRI101 enzymes by CLUSTALW (https://www.genome.jp/tools-bin/clustalw/ (accessed on 14 September 2021)). The secondary structure of TaARL6IP4 was predicted by Jpred 4 (https://www.compbio.dundee.ac.uk/jpred/ (accessed on 14 September 2021)) [56]. Antimicrobial activity was predicted by the Antimicrobial Peptide Database (APD3) (https://aps.unmc.edu/prediction/ (accessed on 14 September 2021)) [57]. Phylogenetic analyses were performed using MEGA 11 software (Mega, Raynham, MA, USA) with the neighbor-joining method.

### 2.5. Subcellular Localization

**Figure 1:**
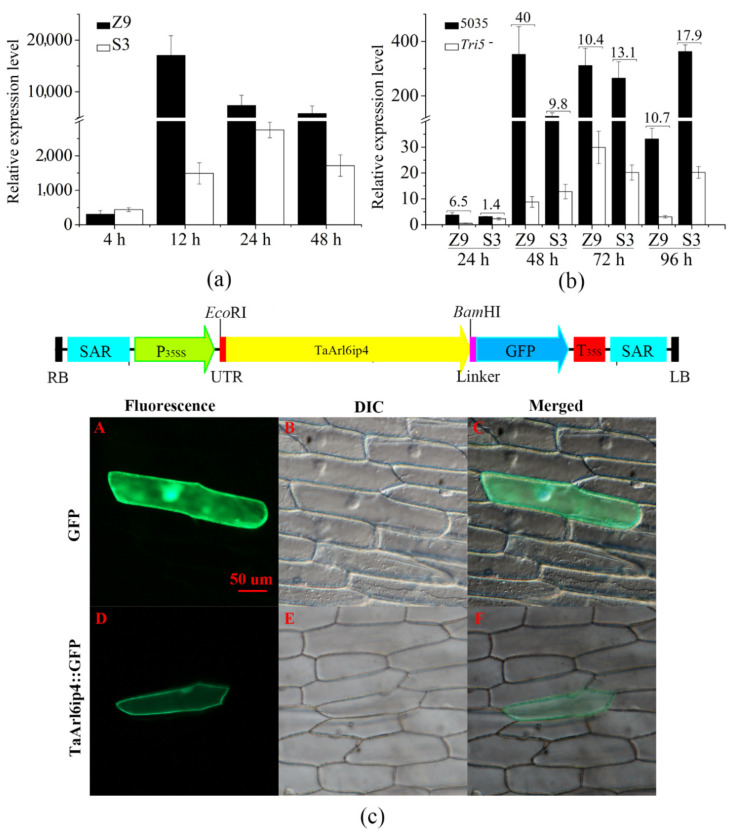
Expression analysis of the DON-responsive wheat gene *TaArl6ip4* in planta. (**a**) Relative expression level analysis of *TaArl6ip4* in Z9 and S3 wheat spikes at 4, 12, 24, and 48 hpi with DON. Z9 represents the wheat cultivar Zhengmai9023, and S3 represents the wheat cultivar Sumai3. (**b**) Relative expression level analyses of the *TaArl6ip4* gene in spikes of two wheat cultivars, Z9 and S3, at 24, 48, 72, and 96 hpi after inoculation with the DON-producing strain *F. graminearum* 5035 (5035) or DON-nonproducing *Tri5* knockout mutant strain (*Tri5¯*). (**c**) Subcellular localization of *TaArl6ip4* in onion epidermal cells. A, GFP fluorescence was detected in plasma membrane, nucleus and cytoplasm of onion epidermal cells expressing GFP alone (control). The differential interference contrast (DIC) and merged images of A are shown in B and C, respectively. D, green fluorescence of TaARL6IP4::GFP fusion construct was observed in the cell membrane and nucleus of onion epidermal cells. DIC and merged images of D are shown in E and F, respectively. The wheat *β-actin* gene was used as a reference gene to calculate the relative gene expression levels of *TaArl6ip4*. All relative gene expression level values are the means ± standard deviations of 20 replicates.

The TaARL6IP4::GFP fusion vector, including a CaMV35S promoter, a NOS terminator, and a green fluorescent protein (GFP) gene [54], was constructed for an onion epidermal cell transient expression assay. In brief, the coding sequence of *TaArl6ip4* was amplified with the *TaArl6ip4*-F4 and *TaArl6ip4*-R4 primer sets (Table S1), containing a 5′ end *EcoR*I site and a 3′ end *BamH*I site with the deleted stop codon. A 5′ UTR sequence was introduced into the vector using the SOE method with the UTR and *TaArl6ip4*-R4a primer sets (Table S1). After double digestion with *EcoR*I and *BamH*I, the resulting fragment was introduced into the binary vector pTRA-GFP. Then, the generated pTRA-TaArl6ip4-GFP (Figure 1c) was introduced into the onion epidermal cells for transient expression by particle bombardment-mediated transformation, and the empty pTRA-GFP vector was used as a control. Particle bombardment was performed as per the manufacturer’s instructions (Bio-rad laboratories, Hercules, CA, USA). The onion epidermis was kept in the dark for 24 h at 25 °C before treatment with a 2 M sucrose solution. Fluorescence microscopic analysis was performed using a Nikon eclipse 90i microscope (Nikon, Tokyo, Japan).

### 2.6. Plant Transformation

The pTRA-TaARL6IP4 vector was constructed with the UTR and *TaArl6ip4*-R4b primer sets (Table S1) using the pTRA-TaArl6ip4-GFP vector as a template. The pTRA-TaARL6IP4 vector was then introduced into *Agrobacterium tumefaciens* strain GV3101 via the electroporation method. *Agrobacterium*-mediated transformation was performed in ‘Columbia’ wild-type *Arabidopsis thaliana* using the floral dip method [58,59]. Transgenic plants were screened for the presence of the *TaArl6ip4* gene on 1/2 MS medium supplemented with 50 µg mL^−1^ of kanamycin, and the positive transgenic lines were identified by PCR and RT-PCR with primers *TaArl6ip4*-F5 and *TaArl6ip4*-R5 (Table S1).

### 2.7. Antifungal Activity

The antifungal activities of the TaARL6IP4 peptide toward five representative fungal pathogens, *F. graminearum*, *Fusarium oxysporum* (*F. oxysporum*), *Alternaria alternata* (*A. alternata*), *Colletotrichum higginsianum* (*C. higginsianum*), and *Sclerotinia sclerotiorumas* (*S. sclerotiorumas*) were investigated as previously described, with slight modifications [60]. Briefly, fresh fungal spore suspensions were harvested and filtered through Whatman #1 paper (Sigma–Aldrich, St. Louis, MO, USA). The final concentrations were adjusted to 5 × 10^4^ spores mL^−1^ in a liquid NB medium. To further validate the growth inhibitory effect of TaARL6IP4 on fungi, the IC_50_ was measured spectrophotometrically at 595 nm in 96-well microtiter plates after incubation of 50 µL of the spore suspensions with 50 µL of twofold serial diluted peptide starting with 200 µM at 28 °C for 24 h.

### 2.8. Microscale Thermophoresis Analysis

Protein samples were labeled with the Monolith NT Protein Labeling Kit RED-NHS (Nanotemper Technologies, München, Germany) at the N-terminal. Reactions were analyzed using premium capillaries containing 16 twofold serial dilutions of DON, starting with 8 mM. In brief, 100 nM labeled GST-tagged TaARL6IP4 or GST protein (control) was incubated with 16 serially diluted DON solutions in a final buffer composed of 10 mM PBS buffer supplemented with 0.05% Triton-100 at 25 °C for 30 min [61]. Fluorescence intensity values from the binding kinetic profiling were measured using the Monolith NT.115 (Nanotemper Technologies, München, Germany). The experimental data were analyzed using GraphPad Prism (GraphPad Software, San Diego, CA, USA). The normalized fraction bound values against the logarithms from the 16 serially diluted DON concentrations were used to generate the dissociation constant *K*_D_. The experiment was performed in triplicate.

### 2.9. Fluorescence Microscopy Analysis

*F. graminearum* 5035 spores were harvested by centrifugation, and after washing three times with PBS buffer, the concentration was then adjusted to 2 × 10^6^ mL^−1^, followed by incubation with PBS and 10 µM of TaARL6IP4 or 20 µM of TaARL6IP4 at 28 °C for 4 h. Then, the treated spores were washed twice with PBS buffer, and propidium iodide (PI) was gently added to a final concentration of 10 µg mL^−1^. The resulting spores were finally fixed on glass slides for 30 min in the dark at 4 °C. A fluorescence microscopic assay was performed using a Nikon eclipse 90i microscope (Nikon, Tokyo, Japan) with an excitation wavelength at 545 nm. PI-treated spores were used for a flow cytometry (Easycyte 8, Millipore, MA, USA) assay as previously described [62], and data were analyzed using FlowJo 7.6 software (BD, Ashland, OR, USA). *F. graminearum* 5035 spores were incubated with 50 µM of FITC-ahx-labeled TaARL6IP4 peptide (GenScript, Piscataway, NJ, USA) and PBS (control) in the dark at 25 °C for 1 h and 18 h. Optical and fluorescence microscopic analyses were carried out at an excitation wavelength of 495 nm.

### 2.10. Transmission Electron Microscopy Assay

*F. graminearum* 5035 spores were harvested by centrifugation, and after washing three times with PBS buffer, the concentration was then adjusted to 2 × 10^6^ mL^−1^. The spores were incubated with TaARL6IP4 at final concentrations of 0, 50, and 100 µM at 28 °C for 4 h followed by washing three times with PBS and then centrifuged at 1000 rpm for 10 min, after which the supernatant was discarded. The pellets were collected and gently fixed in 2.5% glutaraldehyde overnight at 4 °C [63]. The morphological alterations were analyzed by a Tecnai F20 transmission electron microscopy (FEI, Hillsboro, OR, USA) at 200 kV.

### 2.11. DON Tolerance and Fungal Resistance Assays

Seven-day-old T_2_ or T_3_ transgenic and non-transgenic *Arabidopsis thaliana* seedlings were transferred into 24-well tissue culture plates containing 1/2 MS medium supplemented with 0, 5, 10, and 15 µg mL^−1^ of DON. After treatment at 25 °C for 14 days, the root length and fresh weight of both transgenic and non-transgenic lines were calculated. Spray inoculation of seven-week-old T_2_ or T_3_ transgenic *Arabidopsis thaliana* plants and non-transgenic wild-type plants with an *F. graminearum* strain 5035 conidiospore suspension (1 × 10^5^ spores mL^−1^) dissolved in a 0.2% Tween-20 solution was conducted. A 0.2% Tween-20 solution treatment was regarded as the control. Then, the inoculated plants were cultivated in a growth chamber with high relative humidity. The disease index, represented as the *Fusarium-Arabidopsis* disease value (FAD value), was calculated at 7 and 10 days post-inoculation, as previously described [64,65]. Twenty transgenic or non-transgenic seedlings were used for a DON tolerance assay and spray inoculation, respectively. The experiments were performed using 60 seedlings for either transgenic line or wild-type plants.

### 2.12. Southern Blotting and Northern Blotting

Southern blotting was performed as previously described [50]. Genomic DNA was extracted from 30-day-old fresh leaves of Z9 or *Arabidopsis* plants by the CTAB method. A total of 20 µg of wheat genomic DNA was digested with 60 units of *EcoR*I, *Sac*I, or *Hind*III overnight at 37 °C. A total of 5 µg of *Arabidopsis* genomic DNA was digested with 20 units of *Sac*I overnight at 37 °C. Then, the resulting DNA fragments were separated by 0.8% agarose gel electrophoresis and transferred onto nylon membranes (Amersham, Buckinghamshire, UK) and hybridized with an α-[32P]-dCTP-labeled probe of *TaArl6ip4* (nucleotide 1–523) amplified with the *TaArl6ip4*-F3/*TaArl6ip4*-F3 primers (Table S1). For Northern blotting, a total of 20 µg of RNA was isolated from 50 µg mL^−1^ DON- or water-treated spikelets for 24 h. Then, RNA was run on 1.2% agarose/formaldehyde gel, transferred onto nylon membranes, and hybridized with the *TaArl6ip4* probe above. Autoradiography was carried out using a Bas1800 II imaging plate scanner (Fujifilm, Tokyo, Japan).

### 2.13. Statistical Analysis

All data were analyzed using SAS version 9.4 (SAS Institute, Cary, NC, USA) and are presented as the mean ± standard deviation using Student’s *t*-test to measure the significance level at 0.01 or 0.05.

## 3. Results

### 3.1. TaArl6ip4 Encodes an ADP-Ribosylation Factor-Like Protein 6-Interacting Protein 4 in Response to DON

The *Triticum aestivum* gene *TaArl6ip4*, encoding a 73-amino acid peptide, was identified from the DON-treated wheat suspension cell cultures by suppression subtractive hybridization (SSH). A total of 118 prominently up-regulated and 59 down-regulated genes were identified by dot blot assay. A strongly DON-induced gene (accession: OK345034), which encodes ADP-ribosylation factor-like protein 6-interacting protein 4, was named *TaArl6ip4* based on its deduced amino acid sequence sharing a 68–70% identity with ARL6IP4 proteins (accessions: XP_037418635.1 and XP_037418634.1) of *Triticum dicoccoides*. 3′-RACE and 5′-RACE were performed to generate 422-and 468-bp expressed sequence tag (EST) fragments, respectively, with DON-treated Z9 cDNA. A 523-bp-long sequence was assembled that comprised an open reading frame encoding 73 amino acids. Both genomic DNA and cDNA were used to amplify the full-length *TaArl6ip4* (accession: OK345034); the PCR fragment lengths were consistent, which suggests that *TaArl6ip4* has no intron (Figure S1). Phylogenetic analysis of the deduced TaARL6IP4 amino acid sequence, together with ARL6IP4 proteins from 19 plant species, *Arabidopsis* (accession: XP_002887424.2), *Oryza sativa Japonica* Group (XP_015616049.1), *Triticum dicoccoides* (XP_037418635.1 and XP_037418634.1), *Brassica rapa* (XP_033140723.1), *Brassica napus* (XP_013660348.1), *Hibiscus syriacus* (XP_039062773.1 and XP_039057777.1), *Manihot esculenta* (XP_021621638.1), *Helianthus annuus* (XP_035835764.1), *Vitis riparia* (XP_034685127.1), *Rhodamnia argentea* (XP_030536130.1), *Solanum lycopersicum* (XP_004237989.1), *Spinacia oleracea* (XP_021838863.1), *Hevea brasiliensis* (XP_021680875.1 and XP_021675483.1), *Herrania umbratical* (XP_021283269.1), *Tarenaya hassleriana* (XP_010542636.1), *Gossypium arboretum* (XP_017633080.1 and XP_017633081.1), *Nicotiana attenuate* (XP_019240749.1), *Papaver somniferum* (XP_026395539.1, XP_026395540.1, XP_026395541.1, and XP_026395542.1), and *Solanum lycopersicum* (XP_010320203.1), was performed, and the results suggest that TaARL6IP4 and *Triticum dicoccoides* ARL6IP4 may descend from a common ancestor due to their close evolutionary relationship (Figure S2).

To reveal the copy numbers of *TaArl6ip4* in the wheat genome, Southern blotting analysis was carried out with Z9 genomic DNA. The results showed that four bands were observed after non-cutter *EcoR*I or *Sac*I digestion, and eight bands were observed after single-cutter *Hind*III digestion, suggesting the presence of four copies of *TaArl6ip4* in the wheat genome (Figure S3). The Northern blotting results showed that *TaArl6ip4* gene expression was especially activated by DON treatment, while no transcript was detected in the water-treated control (Figure S3).

The relative expression level of the *TaArl6ip4* gene was calculated by qRT-PCR, and the results revealed that the expression of *TaArl6ip4* was significantly up-regulated after DON treatment within 4 h in both Z9 and S3. The expression levels of *TaArl6ip4* quickly increased by up to 305- and 440-fold in Z9 and S3, respectively, at 4 hpi. After 12 h of treatment, the expression level of *TaArl6ip4* dramatically increased up to the maximum level of 17,040-fold at 12 hpi and then declined to 7535-fold at 24 hpi and 5793-fold at 48 hpi in Z9. However, *TaArl6ip4* was most highly induced to 2729-fold at 24 hpi, but then declined to 1715-fold at 48 hpi in S3 (Figure 1a). These results indicate that *TaArl6ip4* was specifically activated by DON stimulation in wheat; the *Fusarium* intermediate susceptible cultivar Z9 showed an earlier and more sensitive response to DON than *Fusarium* resistant cultivar S3. 

To further validate whether DON is a key factor in *TaArl6ip4* gene activation, wheat spikes from Z9 and S3 were inoculated with DON-producing strain *F. graminearum* 5035 and DON-nonproducing mutant strain *Tri5¯* by single floret injection [66]. The results indicate that the expression levels of the *TaArl6ip4* gene induced by *F. graminearum* 5035 were 40, 10, and 11 times higher than those induced by the *Tri5¯*mutant strain in Z9, as well as 10, 13, and 18 times higher than those induced by the *Tri5¯*mutant strain in S3 at 48, 72, and 96 hpi, respectively (Figure 1b). These results suggest that the activation of the *TaArl6ip4* gene is highly relevant to DON and DON-producing *Fusarium* spp.

To reveal the subcellular localization of *TaArl6ip4* in plant cells, a TaARL6IP4::GFP fusion overexpression vector was constructed for the transient expression of *TaArl6ip4* in onion epidermal cells. The results indicate that the green fluorescence signal of the GFP control was observed throughout the whole cell, including the plasma membrane, nucleus, and cytoplasm. However, green fluorescence of the TaARL6IP4::GFP fusion was mainly observed in the cell membrane, as well as a very weak fluorescent signal localized in the nucleus, which was incompatible with the lack of nuclear localization signals (NLS) for *TaArl6ip4* (Figure 1c). In general, *TaArl6ip4* is a plasma membrane- and nucleus-localized gene. Nevertheless, the mechanism of protein transportation of TaARL6IP4 from the cytoplasm into the nucleus remains to be further elucidated.

### 3.2. Sequence Analysis and Functional Prediction of TaARL6IP4

**Figure 2:**
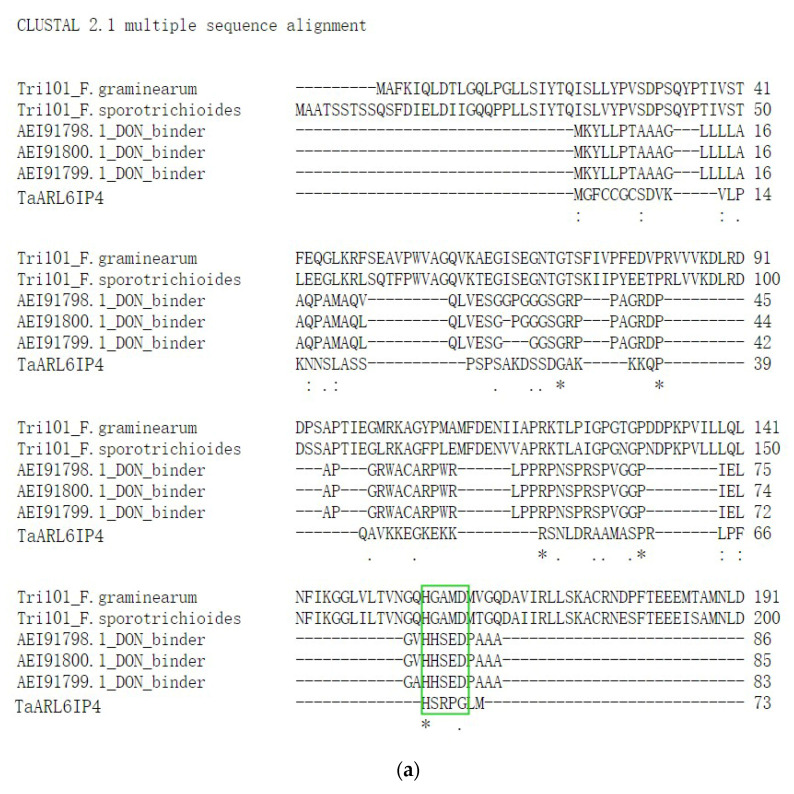

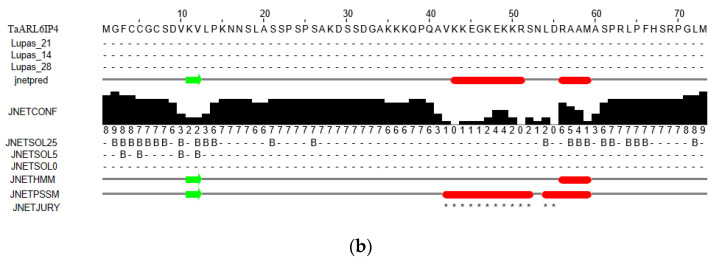
(**a**) ClustalW multiple sequence alignment of TaARL6IP4 with synthesized DON-affinity binders (accessions: AEI91798.1, AEI91799.1, and AEI91780.1) and DON binding/catalysis enzyme TRI101 from *F. graminearum* and *F. sporotrichioides* indicate a highly conserved HXXXD/G motif (highlighted in the green box) and glycine and proline residues (marked by asterisks). (**b**) Prediction of the secondary structure of TaARL6IP4 by Jpred 4 (helices are marked as red tubes and sheets as green arrows).

A previous study revealed that TRI101 is a trichothecene 3-O-acetyltransferase with DON-binding activity, participating in DON secretory pathways in *F. graminearum* and *F. sporotrichioides* [22]. High-affinity heavy chain antibodies (accessions: AEI91798.1, AEI91799.1, and AEI91780.1) toward DON have been reported [23]. Interestingly, sequence alignment of DON-interacting proteins revealed a highly conserved motif, HXXXD/G, involved in DON binding/catalysis [21]. Herein, multiple sequence alignment between TaARL6IP4, three synthesized DON-affinity binders, and TRI101 enzymes was performed by CLUSTAL 2.1. The results showed that the conserved _67_HXXXG_71_ motif and proline and glycine amino acid residues were identified in TaARL6IP4 (Figure 2a). We hypothesize that structural features of TaARL6IP4 might play binding/catalyzing roles in response to DON. The secondary structure of TaARL6IP4 was predicted by Jpred 4, and the results showed that TaARL6IP4 comprises two α helices and one β-sheet structure (Figure 2b). Moreover, *TaArl6ip4* encodes a short peptide comprising 73 amino acids, which was predicted to be an antimicrobial peptide by APD3. The results forecasted that with a protein-binding potential (Boman index) of 2.34 kcal/mol, a molecular weight of 7.78 kDa, a GRAVY of –0.84, and a total net charge of +9.25, the TaARL6IP4 peptide may form alpha helices and may have at least three residues on the same hydrophobic surface, which may interact with membranes and has potential to be an antimicrobial peptide.

### 3.3. TaArl6ip4 Encodes an Antifungal Peptide

**Figure 3:**
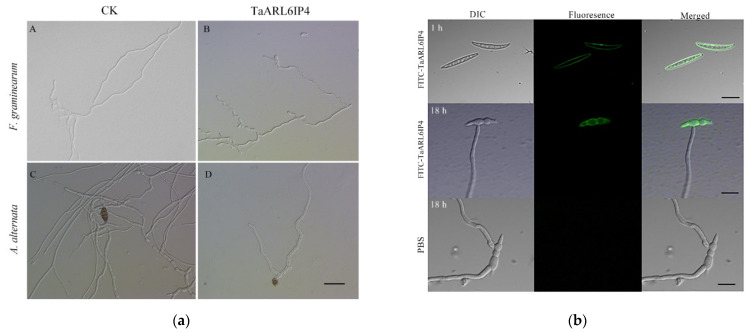
Antifungal activity analysis of TaARL6IP4. (**a**) Growth inhibitory effect of the TaARL6IP4 peptide toward *F. graminearum* and *A. alternata*. (**A**,**B**) *F. graminearum* spores treated with PBS or 50 µM of TaARL6IP4 for 24 h at 28 °C, respectively. (**C**,**D**) *A. alternata* spores treated with PBS or 50 µM of TaARL6IP4 for 24 h at 28 °C, respectively (Bar = 40 µm). (**b**) TaARL6IP4 inhibited the germination of *F. graminearum* spores by targeting the fungal plasma membrane. FITC-ahx-labeled TaARL6IP4 peptide (50 µM) and PBS (control) were used for the treatment of *F. graminearum* 5035 spores in the dark at 25 °C for 1 and 18 h, respectively (Bar = 20 µm).

To verify the hypothesis of whether TaARL6IP4 has antifungal activity, the purified GST-tagged TaARL6IP4 fusion protein was incubated with representative fungal spores for 24 h at 28 °C, and then the inhibition rate was calculated in the form of IC_50_. TaARL6IP4 exhibited a growth inhibitory effect toward *F. graminearum* and *A. alternata* spores (Figure 3a), with IC_50_ values of 22 ± 1.5 µM and 25 ± 2.6 µM, respectively (Table 1). The TaARL6IP4-treated groups showed irregular hyphae with fewer branches, whereas smoother and more regular hyphae were observed in the control (Figure 3a). However, no growth inhibitory effect of TaARL6IP4 toward *F. oxysporum*, *C. higginsianum*, or *S. sclerotiorum* was observed (Table 1).

To clarify the mechanisms behind the antifungal activity of TaARL6IP4 toward *F. graminearum*, an N-terminal FITC-ahx-labeled full-length TaARL6IP4 peptide was synthesized for the fluorescence microscopy analysis. After incubation of FITC-TaARL6IP4 with *F. graminearum* 5035 spores for 1 h, green fluorescence was clearly observed on the plasma membrane of the spores. However, green fluorescence was uniquely observed on the plasma membrane of the spores, but not the mycelia after 18-h treatment with the FITC-labeled TaARL6IP4 peptide (Figure 3b). In addition, the fresh mycelia treated with FITC-labeled TaARL6IP4 for 1 h did not exhibit any fluorescence (data not shown). These results illustrate that TaARL6IP4 can inhibit the germination of *F. graminearum* 5035 spores, rather than the mycelia, by targeting the plasma membrane of the spores.

### 3.4. TaARL6IP4 Exhibits DON Affinity In Vitro

**Figure 4:**
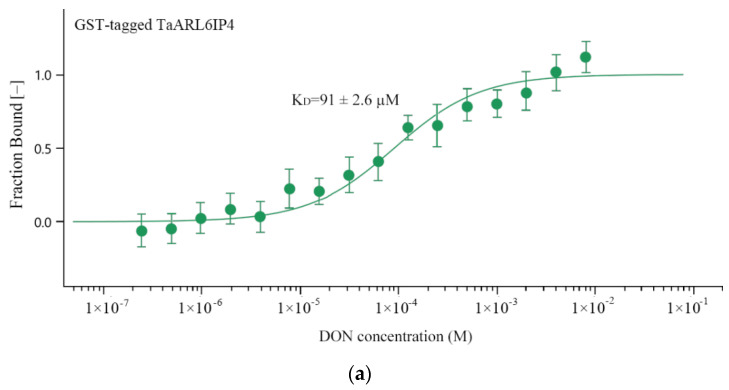

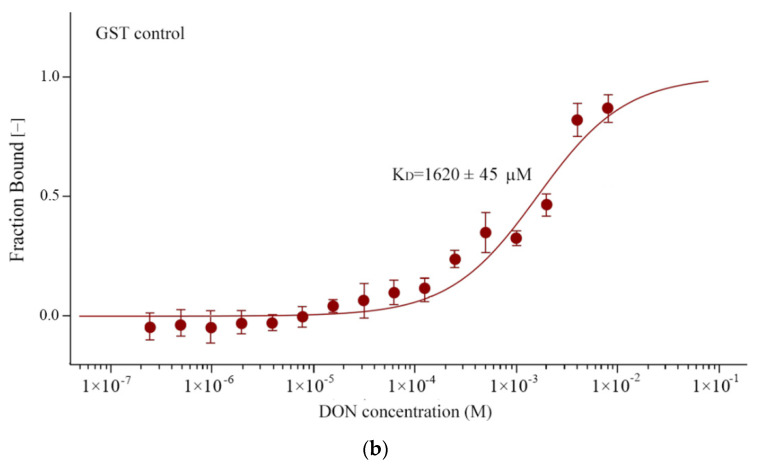
Affinity analysis between TaARL6IP4 and DON in vitro. (**a**) GST-tagged TaARL6IP4 recombinant proteins (100 nM) were separately incubated with 16 twofold serial dilutions of DON, starting with 8 mM. (**b**) GST recombinant proteins (control) (100 nM) were separately incubated with 16 twofold serial dilutions of DON, starting with 8 mM. All values are the means ± standard deviations of three replicates.

Since *TaArl6ip4* is a wheat gene in response to DON, we raised the hypothesis that TaARL6IP4 might be capable of binding DON molecules in plants based on the predicted DON binding/catalysis motif _67_HXXXG_71_. To further validate this assumption, the dissociation constant (*K*_D_) between the TaARL6IP4 peptide and DON was measured by the microscale thermophoresis method in vitro. The results suggest that the TaARL6IP4 peptide exhibited DON affinity with a dissociation constant *K*_D_ of 91 ± 2.6 µM, whereas the control GST protein showed an 18 times lower DON affinity with a *K*_D_ of 1620 ± 45 µM in vitro (Figure 4). These results provide direct experimental evidence of the interaction between TaARL6IP4 and mycotoxin DON.

### 3.5. TaARL6IP4 Disrupts the Membrane Integrity of F. graminearum Spores 

**Figure 5:**
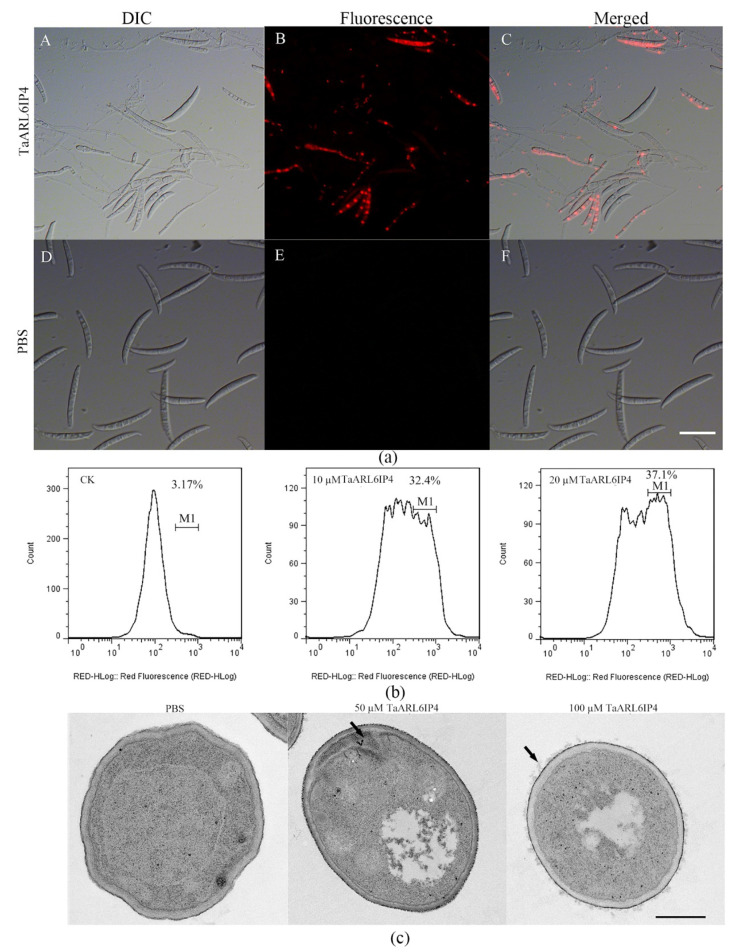
*F. graminearum* membrane disruption and leakage of cytoplasmic materials caused by TaARL6IP4 treatment. (**a**) PI intake assay by fluorescence microscopy. The *F. graminearum* spores (2 × 10^6^ mL^−1^) were incubated with TaArl6ip4 (**A**–**C**) or PBS buffer (**D**–**F**) at 28 °C for 4 h, and the red fluorescence was detected by microscopy (Bar = 20 µm). (**b**) Flow cytometry assay of PI-stained *F. graminearum* spores treated with PBS buffer (control) or 10 or 20 µM of the TaARL6IP4 peptide. The *x*-axis represents the relative fluorescence intensity. M1 = events of necrotic cells with the given fluorescence intensity. (**c**) Morphological change assay by transmission electron microscopy. Spores were treated with PBS (control) or 50 or 100 µM of the TaARL6IP4 peptide at 28 °C for 4 h. Black arrows indicate plasma membrane infoldings and leaked out cytoplasmic materials (Bar = 500 nm).

The plasma membrane is the first biological barrier against extracellular stimulation, comprising a phospholipid bilayer and a transmembrane protein [67]. Well-characterized plant antimicrobial peptides usually act on pathogens and elicit their toxicity by membrane disruption, such as thionins, plant defensins, and lipid transfer proteins (LTPs) [25,27]. To reveal whether TaARL6IP4 disrupts the *F. graminearum* plasma membrane integrity, flow cytometry and a PI intake assay were performed with TaARL6IP4 peptide-treated spores. PI is a red fluorescent nuclear and chromosome dye that cannot penetrate the intact membrane, making it widely used in distinguishing apoptotic and viable cells based on membrane integrity. The PI intake assay results indicated that TaARL6IP4-treated spores were practically stained and showed red fluorescence in the nucleus, whereas PBS treatment did not exhibit any red fluorescence signal in the nucleus (Figure 5a). The flow cytometry results indicated a positive relationship between the concentration of TaARL6IP4 and the degree of membrane damage. The events of necrotic cells with the given fluorescence intensity were 3.17%, 32.4% and 37.1%, corresponding to PBS, 10, and 20 µM of TaARL6IP4 treatment, respectively (Figure 5b). These results indicate that the cell membrane integrity of *F. graminearum* spores was disrupted by TaARL6IP4 in a concentration-dependent manner.

To further validate the disruptive effect of TaARL6IP4 on cell membrane of *F. graminearum* spores, the morphological changes were characterized by a transmission electron microscopy (TEM). The spores treated with PBS exhibited a smooth and integrated cellular morphology with a clear nucleus, cell membrane, cytoplasm, and vacuole. In contrast, plasma membrane infoldings, a disordered cytoplasmic structure, and the leakage of cytoplasmic materials were observed for TaARL6IP4-treated spores (Figure 5c). These results are consistent with the flow cytometry and PI intake assay. Generally, TaARL6IP4 could initially bind to the plasma membrane through interactions with the cell membrane components of *F. graminearum* spores, leading to membrane disruption and leakage of cytoplasmic materials.

### 3.6. TaArl6ip4 Enhances DON Tolerance and FHB Resistance in Arabidopsis 

**Figure 6:**
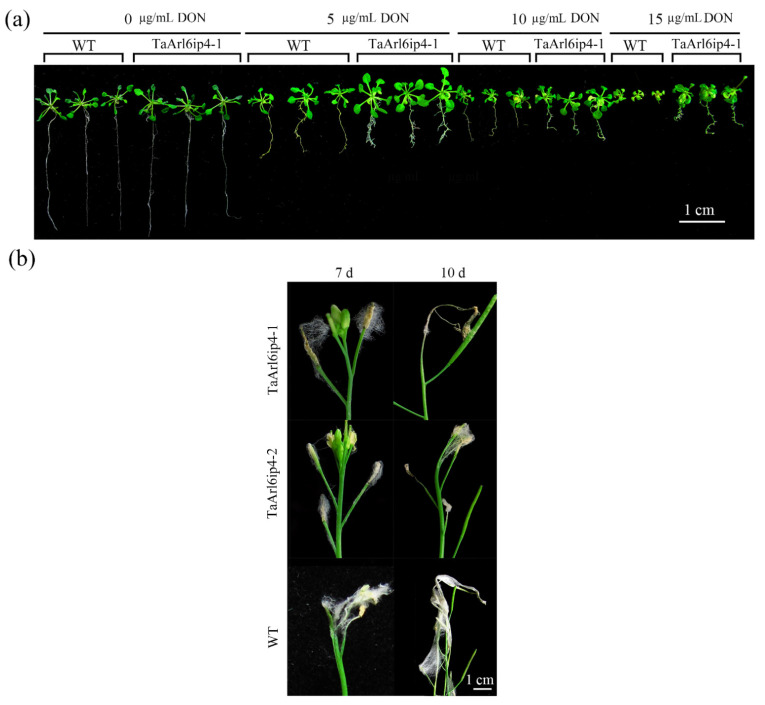
*TaArl6ip4* confers transgenic plants DON tolerance and FHB resistance. (**a**) Phenotypes of T_2_ generation transgenic *Arabidopsis TaArl6ip4*-1 and wild-type *Arabidopsis* (WT) seedlings were germinated in 1/2 MS medium after treatment with 0, 5, 10, or 15 µg mL^−1^ of DON. Photographs were taken at 14 days post inoculation with DON (Bar = 1 cm). (**b**) Phenotypes of florets from T_3_ generation transgenic *Arabidopsis* lines (*TaArl6ip4*-1 and *TaArl6ip4*-2) and wild-type (WT) at 7 and 10 days post inoculation with *F. graminearum* 5035 (Bar = 1 cm). The experiments were performed using 60 plants from either transgenic lines or wild-type genotypes.

To evaluate whether *TaArl6ip4* can improve DON tolerance and *Fusarium* resistance in plants, the full-length ORF of *TaArl6ip4* was introduced into the overexpression vector pTRA-TaARL6IP4 promoted by CaMV 35S to generate transgenic *Arabidopsis* lines. Two independent transgenic lines, *TaArl6ip4-1* and *TaArl6ip4-2* were selected from seven positive T_1_ generation plants for the DON tolerance and *Fusarium* resistance assays. The transgenic lines were identified by PCR and RT-PCR for the presence of the *TaArl6ip4* gene, and its integration into the *Arabidopsis* genome was verified by Southern blotting (Figure S4). As reported, DON exhibited an inhibitory effect on root elongation in plants [17]. The root length elongation was significantly inhibited by DON treatment in both wild-type and transgenic plants. However, the *TaArl6ip4-1* transgenic line showed an apparently longer root length and a greater fresh weight than the wild-type after treatment with 15 µg mL^−1^ of DON (Figure 6a). Therefore, 15 µL mL^−1^ DON was used to evaluate the inhibitory effect of DON on the T_2_ and T_3_ generations of the wild-type and transgenic lines. The transgenic lines showed a longer root length (by 300%) and greater fresh weight (by 70%) on average than wild-type plants (Table 2). These results illustrate that *TaArl6ip4* confers durable DON tolerance for T_2_ and T_3_ generation transgenic plants.

To explore the effect of *TaArl6ip4* against pathogen *F. graminearum* 5035, the *Fusarium–Arabidopsis* disease (FAD) index was calculated among the transgenic and non-transgenic *Arabidopsis* lines. In contrast to the wild-type plants, the FAD indexes were significantly decreased by approximately 41.8% at 7 dpi and 34.7% at 10 dpi in the T_2_ transgenic lines (Table 2). In addition, the FAD indexes were significantly decreased by 40.0% at 7 dpi and by 48.2% at 10 dpi in the T_3_ transgenic lines (Figure 6b and Table 2). Above all, DON-induced wheat *TaArl6ip4* conferred durable resistance to DON and *Fusarium* in transgenic *Arabidopsis*.

## 4. Discussion

Fusarium head blight (FHB) or scab of wheat infected by *Fusarium* pathogens is a devastating plant disease worldwide [2,3,68]. Trichothecene mycotoxin DON produced by *Fusarium* species is one of the most prevalent contaminants in grains and feed, but the biological roles of DON involved in *Fusarium* and wheat interactions are poorly understood. In this study, we cloned and characterized a novel DON-activated wheat ADP-ribosylation factor-like protein 6-interacting protein 4 gene, *TaArl6ip4*, from DON-treated wheat suspension cell culture using the suppression subtractive hybridization (SSH) approach. TaARL6IP4 showed both antifungal activity and DON-binding capacity in vitro, which represents the first report of ARL6IP4 in wheat plants. In addition, the overexpression of *TaArl6ip4* in *Arabidopsis* showed significantly improved DON tolerance and FHB resistance, making it valuable in FHB resistance breeding and basic research on the pathogenic mechanism of *Fusarium* species.

Because of the advantages of resembling the whole plants and easy manipulation in vitro, protoplast cell cultures have become a powerful model system to facilitate metabolic and functional genomics of monocots, particularly in wheat, barley, and maize [69,70,71,72]. Dot blot screening of the SSH library identified 118 clones from the DON-induced cDNA library and 59 clones from the DON-inhibited cDNA library, which were found to be involved in cell rescue and defense and biosynthesis of protein and cellular components. *TaMetRS* was previously reported as a novel wheat methionyl-tRNA synthetase gene in response to DON, which conferred DON tolerance and FHB resistance in plants in a previous study by our laboratory [50]. Our results indicate that *TaArl6ip4* is a specifically activated wheat gene in response to DON; the *Fusarium* intermediate susceptible cultivar Z9 showed an earlier and more sensitive response to DON than the *Fusarium* resistant cultivar S3 (Figure 1a,b).

The trichothecene DON produced by *Fusarium* spp. has previously been reported as a virulence factor allowing infection of plants during fungal pathogenesis. The DON-nonproducing *Tri5* gene knockout *F. graminearum* mutants, which are severely stunted in biosynthesis of trichothecene mycotoxin DON, are less virulent to host plants due to their limited ability to spread in wheat rachis [73,74,75]. Moreover, culmorin (CUL) was recently identified as a *Fusarium* secondary metabolite that showed inhibitory effects on the glucuronidation of DON in vitro [76]. Combinations of CUL and DON showed synergetic phytotoxic effects on wheat, barley and maize. The severity of FHB was positively correlated with the total amount of CUL and DON, while negatively correlated with the CUL/DON ratio [77]. In addition, DON acts as an activator by eliciting classical plant defense responses in wheat, such as the accumulation of reactive oxygen species (ROS), programmed cell death (PCD), and the induction of defense genes, including *PR1.1*, *PR2*, *PR3*, *PR4*, *PR5*, and *PR10* [78]. Therefore, it is of importance to explore cellular targets interacting with DON, thereby advancing our understanding of *Fusarium*–host interactions. To date, several proteins or enzymes that recognize and directly interact with DON have been identified. Trichothecene 3-O-acetyltransferase TRI101 of *F. graminearum* is a well-characterized enzyme involved in the biosynthesis of DON that binds to DON with a kinetic constant *Km* of 11.7 ± 3.5 µM [22]. Three DON-affinity binders have been isolated from the DON-induced phage display library, and their DON-binding abilities have been verified by Phage-ELISA [23]. DOGT1, a UDP-glycosyltransferase (UGT) of *Arabidopsis thaliana*, has been found to be able to detoxify DON by converting DON into the non-toxic DON-3-O-glucoside (D3G) [73]. Subsequently, OsUGT79 from rice and HvUGT13248 from barley have been reported as UGTs exhibiting DON affinity with *Km* values of 0.23 ± 0.06 mM and 3.0 ± 0.6 mM, respectively [79,80]. In the current study, DON-activated *TaArl6ip4*, which encodes a protein with a conserved DON binding/caging motif (_67_HXXXG_71_), was identified by multiple sequence alignment, together with TRI101 and synthesized DON-affinity binders. Recombinant GST-tagged TaARL6IP4 showed DON affinity with a dissociation constant *K*_D_ of 91 ± 2.6 µM in vitro (Figure 4). TaARL6IP4 showed an eightfold lower DON affinity than FgTRI101, as well as approximately 2.5- and 33-times higher DON affinity than OsUGT79 and HvUGT13248, respectively. Accordingly, we hypothesize that *TaArl6ip4* might play a vital role in minimizing the DON toxicity to host plants due to its thousand-fold up-regulated expression level after 12 h (Figure 1a) post-inoculation with DON, regardless of moderate DON affinity at the micromole level.

The subcellular localization results illustrated that *TaArl6ip4* localized to both the plasma membrane and nucleus. Nevertheless, no nuclear localization signal (NLS) was identified in the *TaArl6ip4* gene based on nucleotide sequence analysis (data not shown). Unlike classical nuclear localization signals, there might be other mechanisms involved in TaARL6IP4 transport from the cytoplasm into the nucleus. For instance, human *PTEN* encodes a 403-residue dual-specificity phosphatase without the classic nuclear localization signal. PTEN was reported to be transported into the nucleus by several mechanisms, such as diffusion, cytoplasmic shuttling, cytoplasmic localization signal-dependent export, and monoubiquitylation-dependent import [81]. Meanwhile, Ca^2+^-mediated tyrosil phosphorylation-independent conformational change contributes to the nuclear localization of PTEN [82]. Despite the absence of classical NLS, fibroblast growth factor 2 (FGF2) was found to be able to interact with apoptosis inhibitor 5 (API5) and with its non-classic nuclear localization signal (ncNLS) region, resulting in the transportation of FGF2 from the cytoplasm into the nucleus. However, FGF2 was mainly localized to the cytoplasm when it failed to interact with API5, revealing that API5 may act as a carrier protein for the nuclear localization of FGF2 [83]. In our current study, the mechanisms behind the nuclear localization of *TaArl6ip4* remain to be further uncovered.

Recently, ARF proteins have been reported to play roles in response to biotic and abiotic stresses in plants. The rapid accumulation of *OsARF1* transcripts was detected in response to treatment with hydrogen peroxide (H_2_O_2_), salicylic acid (SA), and the avirulent pathogen *Magnaporthe grisea* strain KJ301 [84]. Constitutive overexpression of *OsARF1* in tobacco showed the spontaneous induction of a mimicked hypersensitive response (HR), activation of pathogenesis-related (PR) genes, accumulation of endogenous SA, and enhanced resistance to *Phytophthora parasitica* [84]. Type III effector HopM1 is responsible for the full virulence of *Pseudomonas syringae* pv tomato strain DC3000 (Pto) to suppress plant defenses by targeting and inducing the degradation of *Arabidopsis* ADP ribosylation factor-guanine nucleotide exchange factor (ARF-GEF) AtMIN7 [85]. A further study showed that effector-triggered immunity (ETI) prevents HopM1-mediated degradation of AtMIN7 in plant cells [86]. Loss-of-function of *ARF1* in *N. benthamiana* has been shown to result in a stunted phenotype and severely interrupted non-host resistance to *P. cichorii*, as well as partially compromising *N* resistance gene-mediated resistance to TMV [87]. ARL6IP4, also known as SRrp37, has been identified as an ARL6-interacting protein using yeast two-hybrid screening [88]. Furthermore, SRrp37 interacts with SC35 and modulates alternative pre-mRNA splicing of adenovirus E1A in vivo with either 5′distal site or 3′ proximal site activation [47]. We hypothesize that TaARL6IP4 may be involved in alternative pre-mRNA splicing of *F. graminearum* in the early FHB infection stage.

Although varieties of plant antifungal peptides have been characterized, DON-activated plant proteins showing antifungal activity have not been reported in wheat thus far. Our current study revealed that wheat TaARL6IP4 has an inhibitory effect on the germination of fungal spores, rather than mycelial elongation, by targeting the fungal plasma membrane. Based on the amino acid sequence-based prediction results from the APD3 database, TaARL6IP4 may form alpha helices and may have at least three residues on the same hydrophobic surface, which may interact with membranes and has potential to be an anti-microbial peptide. Our results showed that TaARL6IP4 can inhibit the germination of *F. graminearum* and *A. alternata* at IC_50_ values of 22 ± 1.5 µM and 25 ± 2.6 µM, respectively. However, no antifungal activity toward *F. oxysporum*, *C. higginsianum*, or *S. sclerotiorum* was observed (Table 1). In addition, the mechanism of antimicrobial peptide interaction with microbes is believed to be associated with cell apoptosis due to membrane disruption, followed by the attack of intracellular targets [25,89]. In this study, the antifungal mechanism of TaARL6IP4 was preliminarily analyzed by fluorescence microscopy and a TEM assay. The results showed that TaARL6IP4 can uniformly interact with the plasma membrane of *F. graminearum* spores within 1 h (Figure 3b). The cell membranes were mostly disrupted after treatment with TaARL6IP4 for 4 h, along with plasma membrane infoldings and the leakage of cytoplasmic materials (Figure 5c). Plant AMPs are usually a family of small peptides with a broad antifungal spectrum and can be utilized as promising resistant resources for the protection of crops by transgenic methods. The antifungal peptide AlfAFP derived from *Medicago sativa* seeds has been reported to be capable of conferring resistance to the pathogen *Verticillium dahliae* in transgenic potato plants [90,91]. Heterologous constitutive expression of wheat puroindoline genes *pinA* and/or *pinB* in rice has been shown to significantly enhance the resistance to *Magnaporthe grisea* and *Rhizoctonia solani* [92]. Heterologous expression of potato antimicrobial peptide SN1 by the particle bombardment-mediated approach improves the transgenic wheat resistance to soil-borne fungus *Gaeumannomyces graminis* var. *tritici (Ggt)* [93]. In our study, transgenic *Arabidopsis* overexpressing *TaArl6ip4* significantly promoted DON tolerance and FHB resistance compared to the wild-type plants. 

In conclusion, we identified and characterized the novel wheat gene *TaArl6ip4* in response to DON stimulation. The expression of *TaArl6ip4* was especially triggered by DON treatment in wheat spikes. The TaARL6IP4 peptide exhibited an inhibitory effect on the germination of *F. graminearum* and *A. alternata* spores by targeting the plasma membrane, resulting in cell lysis due to the disruption of the fungal plasma membrane and the leakage of cytoplasmic materials. Furthermore, in vitro binding analysis revealed that TaARL6IP4 has a moderate DON binding affinity with a *K*_D_ of 91 ± 2.6 µM. Moreover, *TaArl6ip4* conferred transgenic plants enhanced DON tolerance and resistance to *Fusarium* pathogens. Finally, our findings suggest that *TaArl6ip4* may play a key role in wheat–pathogen interactions and can be utilized as a potential genetic source for FHB resistance breeding.

## Figures and Tables

**Table 1:** Antifungal analysis of TaARL6IP4 against representative fungi.

Fungi	IC_50_
*F. graminearum* 5035	22 ± 1.5 µM
*A. alternata*	25 ± 2.6 µM
*F. oxysporum*	>200 µM
*C. higginsianum*	>200 µM
*S. sclerotiorum*	>200 µM

All values are the means ± standard deviations of three replicates.

**Table 2:** DON tolerance and *Fusarium–Arabidopsis* disease (FAD) assays of TaArl6ip4 transgenic *Arabidopsis*.

Genotype	Root Length (mm)		Fresh Weight (mg)		Disease (FAD)			
	T_2_	T_3_	T_2_	T_3_	T_2_		T_3_	
	14 dpi	14 dpi	14 dpi	14 dpi	7 dpi	10 dpi	7 dpi	10 dpi
*TaArl6ip4-1*	17.00 ± 1.63 ^a^	19.12 ± 2.95 ^a^	6.71 ± 1.27 ^a^	7.23 ± 1.39 ^a^	6.48 ± 2.17 ^a^	9.15 ± 1.93 ^a^	4.76 ± 1.73 ^a^	5.10 ± 2.20 ^a^
*TaArl6ip4-2*	14.36 ± 2.38 ^a^	17.89 ± 1.69 ^a^	6.15 ± 1.57 ^a^	6.77 ± 1.23 ^a^	7.32 ± 1.87 ^a^	10.10 ± 2.31^a^	5.80 ± 2.07 ^a^	6.20 ± 2.07 ^a^
WT	3.72 ± 0.55	4.40 ± 0.71	3.17 ± 0.74	4.45 ± 0.89	11.86 ± 2.32	14.74 ± 2.16	7.93 ± 1.02	9.85 ± 1.87

All values are the means ± standard deviations of 60 seedlings for each transgenic line. Student’s *t*-tests were used to evaluate the data: ^a^ Statistical significance at *p* < 0.01.

## Data Availability

The data presented in this study are available upon request from the corresponding authors.

## Supplementary Materials

The following are available online at https://www.mdpi.com/article/10.3390/jof7110941/s1, Figure S1: PCR amplification of *TaArl6ip4* using of 3′-RACE and 5′-RACE from the wheat Zhengmai9023 cDNA. Figure S2: Phylogenetic tree analysis of deduced TaARL6IP4 amino acid sequence together with ARL6IP4 proteins from 19 plant species. Figure S3: Southern blotting and Northern blotting analysis of the *TaArl6ip4* gene in wheat. Figure S4: Molecular characterization of the *TaArl6ip4* T_2_ generation transgenic lines in *Arabidopsis* plants. Table S1: Primer sequences used in this study.
