# Supplementary material for: A Novel Deoxynivalenol-Activated Wheat Arl6ip4 Gene Encodes an Antifungal Peptide with Deoxynivalenol Affinity and Protects Plants against Fusarium Pathogens and Mycotoxins
Source: J Fungi (Basel). 2021 Nov 6;7(11):941. doi: 10.3390/jof7110941 (PMC8618893; doi:10.3390/jof7110941)
Supplement: Supplementary file 1 [file jof-07-00941-s001.zip › jof-1434079-supplementary/Supplementary materials.pdf]

Supplementary Materials

Supplementary materials for the article "A Novel Deoxynivalenol-Activated Wheat *Arl6ip4* Gene Encodes An Antifungal Peptide with Deoxynivalenol Affinity and Protects Plants against *Fusarium* Pathogens and Mycotoxins"

Gang Liu [1,2,†], Dong-Yun Zuo [1,2,3†], Peng Yang [1,2], Wei-Jie He [1,2], Zheng Yang [1,2], Jing-Bo Zhang [1,2], Ai-Bo Wu [5], Shu-Yuan Yi [1,2,6], He-Ping Li [1,4], Tao Huang [1,4,*] and Yu-Cai Liao [1,2,*]

* Correspondence 1: Yu-Cai Liao, College of Plant Science and Technology, Huazhong Agricultural University, Wuhan 430070, China.

E-mail: yucailiao@mail.hzau.edu.cn

* Correspondence 2: Tao Huang, College of Life Science and Technology, Huazhong Agricultural University, Wuhan 430070, China.

E-mail: hhttao@mail.hzau.edu.cn

A TABLE OF CONTENTS

Figure S1 PCR amplification of *TaArl6ip4* using of 3′-RACE and 5′-RACE from the wheat Zhengmai9023 cDNA.

Figure S2 Phylogenetic tree analysis of deduced TaARL6IP4 amino acid sequence together with ARL6IP4 proteins from 19 plant species.

Figure S3 Southern blotting and Northern blotting analysis of the *TaArl6ip4* gene in wheat.

Figure S4 Molecular characterization of *TaArl6ip4* T_2 generation transgenic lines in *Arabidopsis* plants.

Table S1 Primer sequences used in this study.

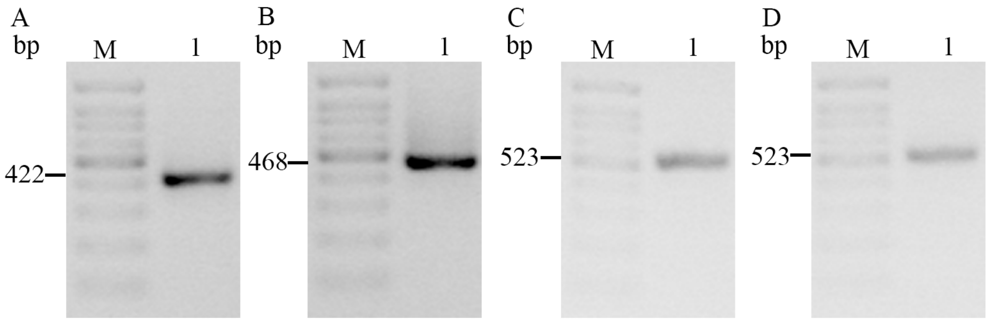

Figure S1 PCR amplification of *TaArl6ip4* using of 3′-RACE and 5′-RACE from the wheat Zhengmai9023 cDNA.

Lane M: Marker 3000 bp; A, lane 1 represents the product of *TaArl6ip4* 3′-RACE; B, lane 1 represents the product of *TaArl6ip4* 5′-RACE; C, lane 1 represents the full-length *TaArl6ip4* PCR product using cDNA as a template; D, lane 1 represents the full-length *TaArl6ip4* PCR product using genomic DNA as a template. All the PCR products were electrophoresed on 1% agarose gels.

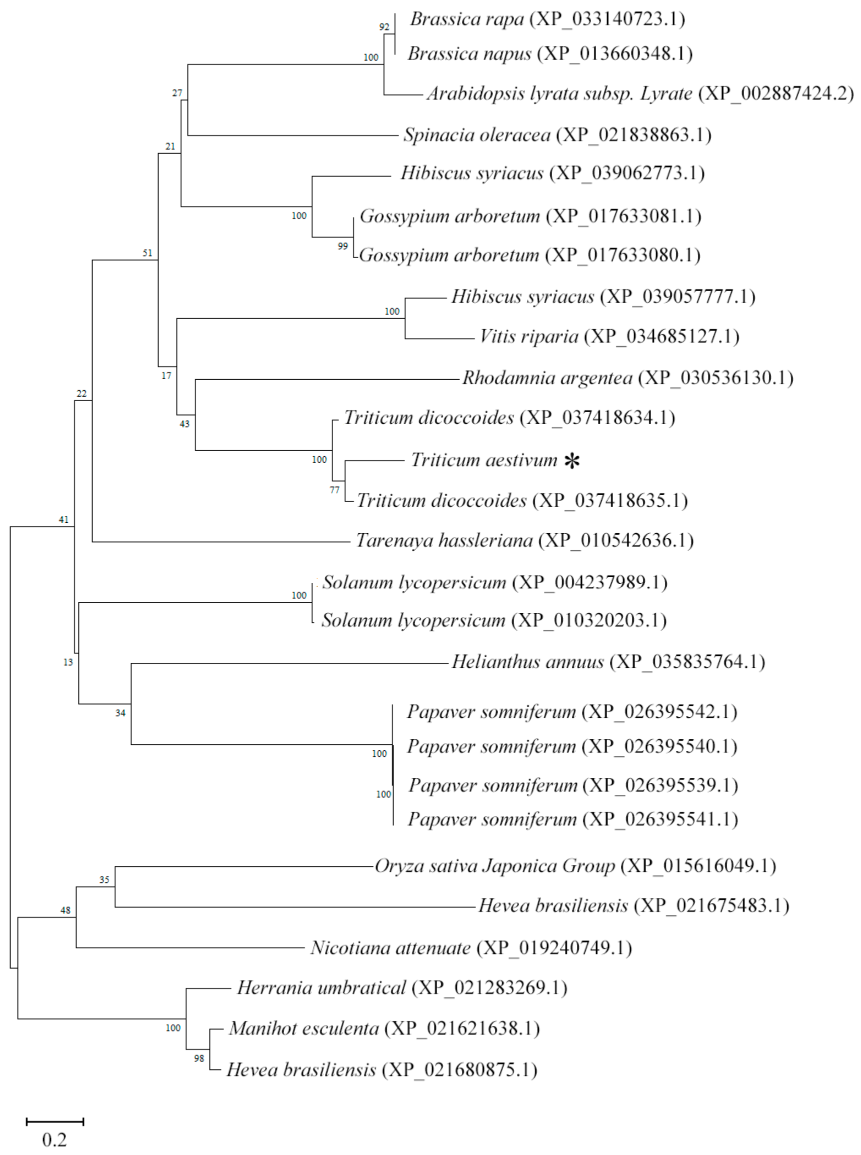

Figure S2 Phylogenetic tree analysis of deduced TaARL6IP4 amino acid sequence together with ARL6IP4 proteins from 19 plant species. *Arabidopsis* (Accession: XP_002887424.2), *Oryza sativa Japonica* Group (XP_015616049.1), *Triticum dicoccoides* (XP_037418635.1, XP_037418634.1), *Brassica rapa* (XP_033140723.1), *Brassica napus* (XP_013660348.1), *Hibiscus syriacus* (XP_039062773.1, XP_039057777.1), *Manihot esculenta* (XP_021621638.1), *Helianthus annuus* (XP_035835764.1), *Vitis riparia* (XP_034685127.1), *Rhodamnia argentea* (XP_030536130.1), *Solanum lycopersicum* (XP_004237989.1), *Spinacia oleracea* (XP_021838863.1), *Hevea brasiliensis* (XP_021680875.1, XP_021675483.1), *Herrania umbratical* (XP_021283269.1), *Tarenaya hassleriana* (XP_010542636.1), *Gossypium arboretum* (XP_017633080.1, XP_017633081.1), *Nicotiana attenuate* (XP_019240749.1), *Papaver somniferum* (XP_026395539.1, XP_026395540.1, XP_026395541.1, XP_026395542.1) and *Solanum lycopersicum* (XP_010320203.1). Amino acid sequences of ARL6IP4 proteins from different plant species were used to generate the phylogenetic tree using MEGA 11 with the neighbor-joining method. The '*' represents amino acid sequence derived from coding sequence of *Triticum aestivum TaArl6ip4* (Accession: OK345034) in the current study.

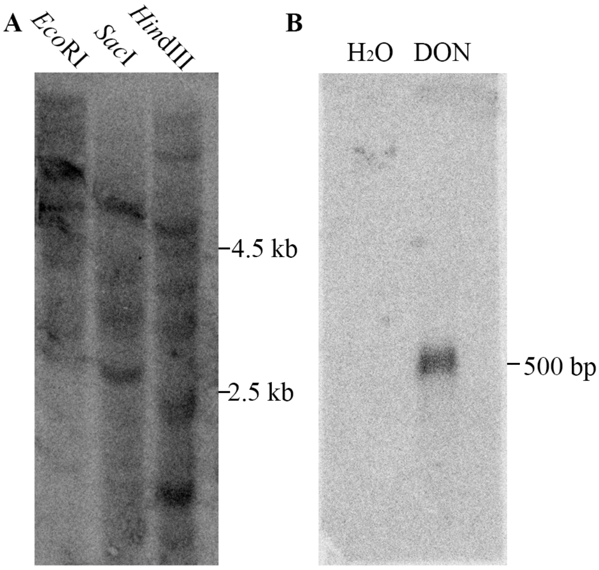

Figure. S3 Southern blotting and Northern blotting analysis of the *TaArl6ip4* gene in wheat.

A, Southern blotting analysis of the *TaArl6ip4* gene expression in wheat. The Zhengmai9023 genomic DNA was digested with *Eco*RI, *Sac*I and *Hind*III, electrophoresis was performed on 0.8% agarose gel and transferred onto nylon membranes and hybridized with a *TaArl6ip4* probe. B, Northern blotting analysis of the *TaArl6ip4* gene expression in wheat. Total RNAs were extracted from water-treated (control), and 50 μg ml^{-1} DON-treated wheat spikelets at 24 hpi, 20 μg RNAs were run on 1.2% agarose/formaldehyde gel and transferred onto nylon membranes and hybridized with a *TaArl6ip4* probe.

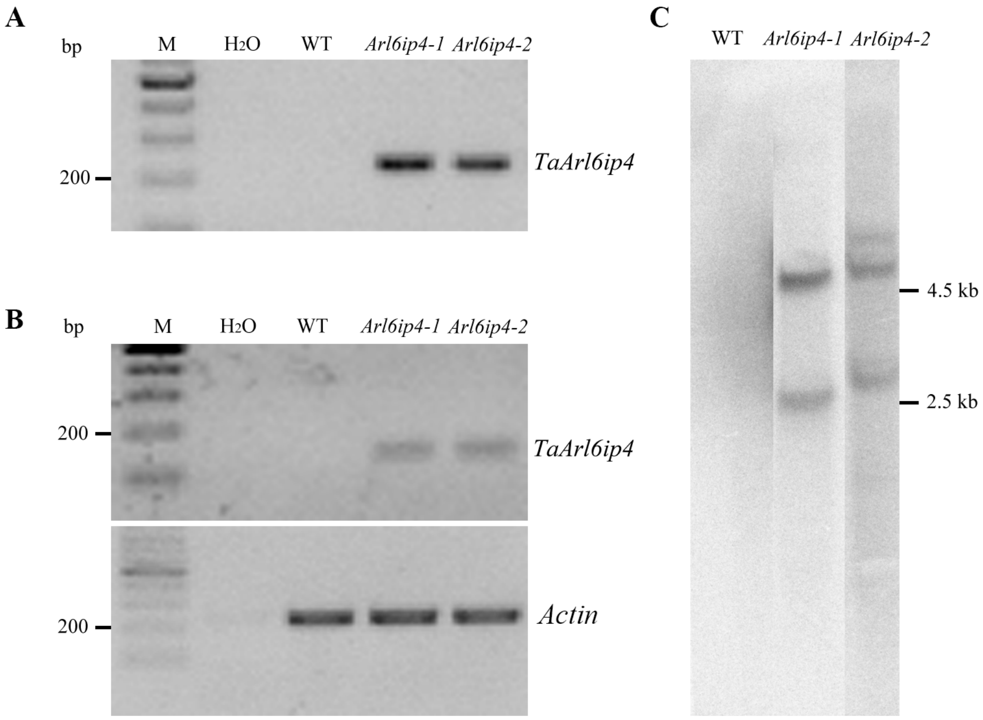

Figure S4 Molecular characterization of *TaArl6ip4* T_2 generation transgenic lines in *Arabidopsis* plants

A, PCR analysis of the transgenic plants *TaArl6ip4-1* and *TaArl6ip4-2* with gene specific primers (UTR/*TaArl6ip4*-R4-b); B, RT-PCR analysis of the transgenic plants *TaArl6ip4-1* and *TaArl6ip4-2* with primers (*TaArl6ip4*-F5/ *TaArl6ip4*-R5), the constitutively expressed *β-actin* gene (*Actin*-F/ *Actin*-R) was used as a reference gene; C, Southern blotting analysis of the transgenic plants *TaArl6ip4-1* and *TaArl6ip4-2*.

Table S1 Primer sequences used in this study

Primers	Sequences (5'-3')
5'RACE-F1	CTAATACGACTCACTATAGGGCAAGCAGTGGTATCAACGCAGAGT
5'-RACE R1	TCTTCAGCGGAACAACCGAGGCAAA
5'RACE-F2	AAGCAGTGGTATCAACGCAGAGT
5'-RACE R2	CCGGGCGTAAGCTAATAAGAGAAC
3'-RACE F1	AGGGACGGGGAAATTTTAGTGGTGTA
3'RACE R1	GCTGTCAACGATACGCTACGTAACG
3'-RACE F2	ATGGGGTTTTGCTGTGGGTGTTC
3' RACE-R2	CGCTACGTAACGGCATGACAGTG
TaArl6ip4-F3	ACATGGGGACAGAAACACATCAAGCA
TaArl6ip4-R3	GGATCAACCGACCGTCCATGAATAA
UTR	CGCGAATTCACAACACAAATCAGATTTATAGAGAGATTTATAAAAAAA AAAAA
TaArl6ip4-F4	TTATAAAAAAAAAAAAATGGGGTTTTGCTGTG
TaArl6ip4-R4a	AGCGGATCCCATTAGGCCGGGTCGA
TaArl6ip4-R4b	ATGCGGATCCTTACATTAGGCCGGGTCGAGAA
TaArl6ip4-F5	GCTGTGGGTGTTCGGATGTC
TaArl6ip4-R5	TTTCCCTTCCTTCTTTACGG
Actin-F	CAGCAATGTATGTCGCAATC
Actin-R	TAGCATGAGGAAGCGTGTAT
TaArl6ip4-F6	TCCAGGGGCCCCTGGGATCCATGGGG TTTTGCTGTGGGTGTT
TaArl6ip4-R6	CTCGAGTCGACCCGGGAATTCTTACAT TAGGCCGGGTCGAGAATG
